# Maize Transposable Elements *Ac*/*Ds* as Insertion Mutagenesis Tools in *Candida albicans*

**DOI:** 10.1534/g3.117.300388

**Published:** 2018-01-29

**Authors:** Kevin Mielich, Ella Shtifman-Segal, Julia C. Golz, Guisheng Zeng, Yue Wang, Judith Berman, Reinhard Kunze

**Affiliations:** * Institute of Biology, Dahlem Centre of Plant Sciences, Free University pf Berlin, 14195, Germany; † Department of Molecular Microbiology and Biotechnology, George Wise Faculty of Life Sciences, Tel Aviv University, Ramat Aviv 69978, Israel; ‡ Institute of Molecular and Cell Biology, Agency for Science, Technology and Research, 138673, Singapore

**Keywords:** *Candida albicans*, *Zea mays*, *Activator/Dissociation*, transposon tagging, insertion mutagenesis

## Abstract

In nonmodel systems, genetic research is often limited by the lack of techniques for the generation and identification of gene mutations. One approach to overcome this bottleneck is the application of transposons for gene tagging. We have established a two-element transposon tagging system, based on the transposable elements *Activator* (*Ac*)/*Dissociation* (*Ds*) from maize, for *in vivo* insertion mutagenesis in the fungal human pathogen *Candida albicans*. A nonautonomous *Ds* transposon carrying a selectable marker was constructed into the *ADE2* promoter on chromosome 3 and a codon usage-adapted *Ac* transposase gene was inserted into the neutral *NEUT5L* locus on chromosome 5. In *C. albicans* cells expressing the transposase, the *Ds* element efficiently excised and reintegrated elsewhere in the genome, which makes the *Ac*/*Ds* transposons promising tools for saturating insertion mutagenesis in clinical strains of *C. albicans*.

The autonomous transposable element *Activator* (*Ac*) from *Zea mays*, originally discovered as the first transposon by McClintock (1951), is one of three prototypic elements of the hAT superfamily of “cut-and-paste” transposons. Soon after its discovery, the potential of *Ac* and its nonautonomous derivatives, termed *Dissociation* (*Ds*), for gene tagging and insertion mutagenesis in maize and heterologous plants was recognized (Walbot 1992). *Ac*/*Ds* elements are convenient to use in heterologous plants because the single *Ac*-encoded transposase protein (TPase) is sufficient for transposition, the transposon ends with the essential TPase-binding sites are only ∼240 bp in length, and the nonautonomous *Ds* elements can carry > 15 kb of cargo DNA. *Ac/Ds* has been used in > 20 higher plants, among them model plants and agronomically important species [reviewed in Kunze and Weil (2002)]. *Ac*/*Ds* elements tend to transpose into genetically linked sites [Dooner and Belachew 1989; Vollbrecht *et al.* 2010; reviewed in Lazarow *et al.* (2013)], but they do not have the target sequence preference exhibited by other transposons (Spradling *et al.* 2011). More recently, the focus of insertion mutagenesis approaches has shifted from single-gene tagging toward the generation of large insertion mutant populations. The use of transposons for this purpose is highly attractive for the study of plants because transposons enable the formation of a large number of insertion mutations in plant species where ballistic or *Agrobacterium tumefaciens*-based Transfer DNA (T-DNA) transformation is inefficient [reviewed in Lazarow *et al.* (2013)]. *Ac*/*Ds* transposons also function in vertebrate animals (Emelyanov *et al.* 2006; Quach *et al.* 2015; Vrljicak *et al.* 2016) as well as in the budding yeast *Saccharomyces cerevisiae* (Weil and Kunze 2000). Recently, *Ac*/*Ds* was employed for saturated transposon mutagenesis in *S. cerevisiae*, facilitating the identification of conditionally essential genes and identifying important functional protein domains (Michel *et al.* 2017). An important advantage is the facility of generating libraries of insertion mutations in mutant strain backgrounds. A similar approach, using the hAT element Hermes from the house fly (*Musca domestica*), achieved saturated transposon insertion mutagenesis in the fission yeast *Schizosaccharomyces pombe* (Guo *et al.* 2013).

Transposable elements in eukaryotic genomes are mostly inactive and transpose only rarely. This relative stability of transposon insertions is due to epigenetic silencing, the low activity of the TPase, as well as post-translational negative autoregulation, such that increased TPase levels result in reduced transposition frequency [reviewed by Bire *et al.* (2013)]. This phenomenon, termed TPase “overexpression inhibition,” presumably underlies the “inverse dose effect” of *Ac*/*Ds* first discovered by McClintock (1951) and later also reported in heterologous plants (Scofield *et al.* 1993, Heinlein *et al.* 1994). The naturally low activity of transposons limits the efficiency of transposon mutagenesis experiments; hyperactive TPase variants that catalyze more frequent transposition reactions have increased the efficiency of transposon mutagenesis with several prokaryotic and eukaryotic transposons (Goryshin and Reznikoff 1998; Lampe *et al.* 1999; Beall *et al.* 2002; Zayed *et al.* 2004; Mátés *et al.* 2009; Yusa *et al.* 2011; Lazarow *et al.* 2012). For example, *Ac*TPase4x, a hyperactive *Ac* TPase, catalyzes a higher *Ds* excision frequency compared to the wild-type *Ac*TPase [100-fold higher in budding yeast and sixfold higher in *Arabidopsis thaliana* (Lazarow *et al.* 2012)].

Here, we asked if the maize *Ac*/*Ds* transposon system could be applied to nonmodel micro-organisms more distantly related to the model budding and fission yeasts. The commensal ascomycete *Candida albicans* is the most common fungal pathogen of humans (Kim and Sudbery 2011) and differs in several genetic and morphological features from *S. cerevisiae*. In addition, *C. albicans* diverges from the universal genetic code at one codon: CTG encodes serine instead of leucine, which complicates the design of heterologous molecular genetic systems. Furthermore, *C. albicans* was long thought to be an obligate diploid with the ability to undergo a “parasexual cycle” between generations without conventional meiosis from diploid to tetraploid and back [reviewed by Noble and Johnson (2007)]. However, mating-competent haploid cells were discovered recently (Hickman *et al.* 2013). Haploids appear to arise via a concerted chromosome loss mechanism rather than meiosis. The initial isolates grew slower than diploids and were relatively unstable, reverting to “autodiploids” with high frequency (Hickman *et al.* 2013). Here, we describe a haploid strain selected for its faster growth rate and use it to test the design of an inducible two-component *Ac*/*Ds* transposon system for *C. albicans*, consisting of a codon-adapted hyperactive TPase gene and a nonautonomous *Ds* element carrying a selection marker. We demonstrate that the TPase mobilizes the *Ds* element and that the *Ds* excision footprint sequences exhibit subtle differences compared to those seen in *S. cerevisiae* and plants.

## Materials and Methods

### Construction of *Ac/Ds* components

**Figure 1:**
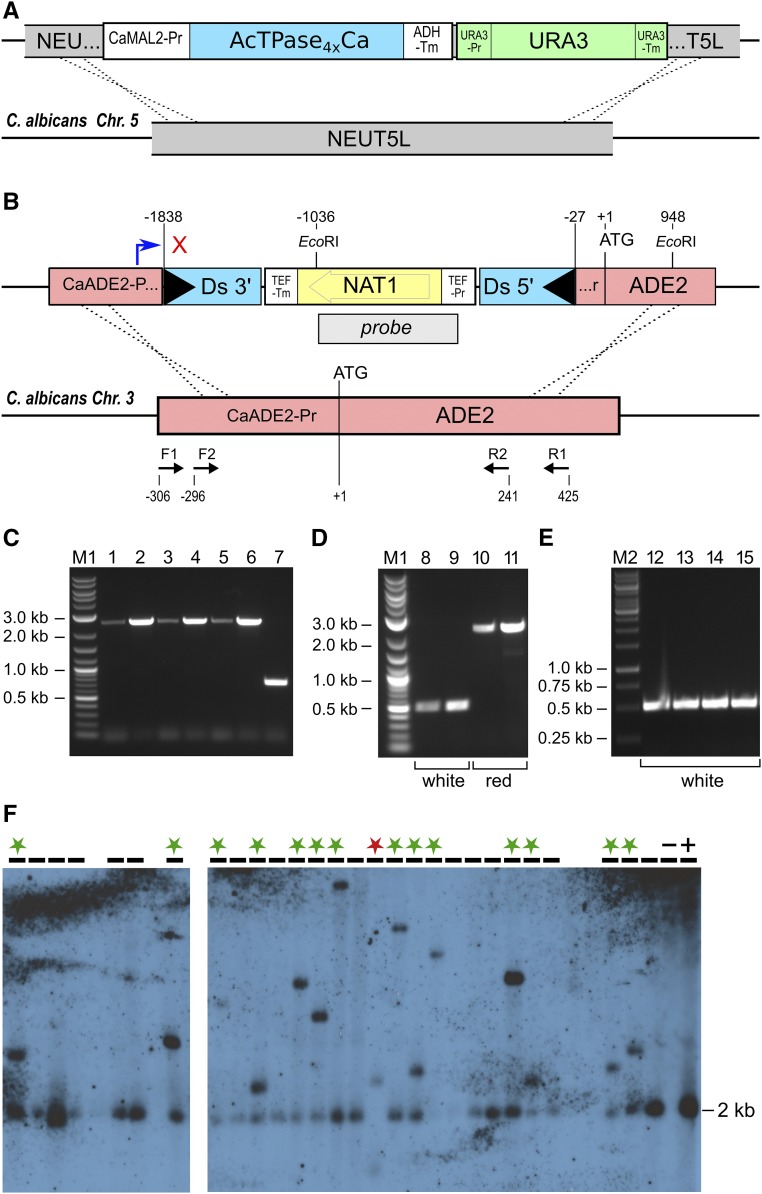
*Ac*/*Ds* (*Activator*/*Dissociation*) transposition in *C. albicans*. (A) The transposase expression construct consists of the *Ac*TPase4xCa ORF (blue) inserted between the *CaMAL2* promoter and the *CaADH1* terminator, flanked by the *CaURA3* gene (green), and inserted into the *NEUT5L* locus for homologous recombination into that locus on *C. albicans* chromosome 5. (B) The *ade2*::*Ds-NAT1* cassette consists of a nonautonomous mini*Ds* transposon (blue) that carries a *NAT1* selectable marker gene (yellow). The transposon is inserted into the 5′-UTR of the *CaADE2* gene (red). This cassette was inserted by homologous recombination into the *ADE2* gene on chromosome 3. The blue bent arrow indicates the transcriptional start site of the *CaADE2* promoter and the red cross symbolizes transcriptional interruption by the *Ds-NAT1* transposon. TEF-Pr, *TEF* promoter; TEF-Tm, *TEF* terminator; Black triangles, *Ds* terminal inverted repeats; Probe, hybridization probe used for DNA gel blot analysis shown in (F); F1/2 and R1/2, *CaADE2* primers used for PCR analyses shown in (C–E). (C) PCR products obtained with primers F1 and R1 on genomic DNA from KMY100 colonies after four successive cell passages (lane 1, passage 0; lane 2, passage 1; lane 3 passage 2; lane 4, passage 3; and lanes 5–6, passage 4) and from the progenitor GZY896 strain (lane 7). (D) PCR products obtained with primers F2 and R2 on genomic DNA from white (lanes 8–9) and red (lanes 10–11) KMY103G1 colonies grown on inducing SDC (synthetic defined minimal) plates (+ adenine + maltose). (E) PCR products obtained with primers F2 and R2 on genomic DNA from white KMY103G1 colonies (lanes 12–15) grown on inducing SDC plates (+ adenine and + maltose). These PCR products were cloned and sequenced for *Ds* excision footprint analyses. M1, New England Biolabs Quick-Load 2-Log DNA Ladder and M2, Thermo Fisher Scientific GeneRuler 1 kb DNA Ladder. (F) DNA gel blot analysis of *Ds-NAT1* element distribution in *ADE2* revertant clones. Genomic DNA from KMY103G1 *ADE2* revertant colonies was *Eco*RI-digested, size-fractionated, blotted, and hybridized with a *NAT1*-probe. Samples in the indicated lanes were evaluable. Lanes marked with green asterisks: an additional band hybridized, indicating *Ds-NAT1* excision and reintegration. Unmarked lanes: only the 2-kb fragment of the *ade2*::*Ds-NAT1* allele hybridized. Lane marked with red asterisk: two novel bands indicate excision and reintegration of both *Ds-NAT1* elements. Lane marked “−”: GZY896 DNA (negative control). Lane marked “+”: KMY100 DNA (positive control).

The codon-adapted *Ac*TPase4xCa open reading frame (Supplemental Material, Figure S1 in File S1) was constructed by fusing three *de novo*-synthesized DNA fragments (Thermo Fisher Scientific GENEART GmbH, Regensburg, Germany) and cloned into pJET1.2 via the CloneJET kit (Thermo Fisher Scientific). The *CaMAL2* promoter (Backen *et al.* 2000), amplified from pKB2019 with primers oMAL2-F and oMAL2-R, and the *CaADH1* terminator, amplified from pMG2120 (Gerami-Nejad *et al.* 2012) with primers oADHT-F and oADHT-R, were also ligated into pJET1.2. The *Ac*TPase4xCa expression cassette was assembled by fusing the promoter, *Ac*TPase4xCa-coding region, and terminator in pJET1.2. From this plasmid, the *Ac*TPase4xCa expression cassette was excised with *Eco*91I and ligated into *Eco*91I-linearized pDUP5 (Gerami-Nejad *et al.* 2013), yielding pKM300 (Figure 1A, top line).

To construct a nonautonomous *miniDs* element with a selectable marker inserted into the 5′-UTR of the *C. albicans ADE2* gene, the *miniDs* from pBBDsXho (Laufs *et al.* 1990) was excised with *Sal*I and cloned into pUC19. The resulting plasmid was pRK86. The nourseothricin acetyl N-transferase gene *NAT1*, under the control of the *TEF* promoter, was amplified from pDUP3 (Gerami-Nejad *et al.* 2013) with the primers oTEFmut-F and oTEFmut-R, and the PCR product was digested with *Sal*I and ligated into *Xho*I-linearized, dephosphorylated pRK86 to form pRK86-*NAT1*, carrying the transposable element *Ds-NAT1*. A 528-bp segment of *C. albicans* chromosome 3, centered around an artificial *Sal*I site 24-bp upstream of the *ADE2* start codon, was *de novo* synthesized (Thermo Fisher Scientific) and ligated into pJET1.2. The resulting plasmid, pRK401, was linearized with *Sal*I and dephosphorylated with FastAP (Thermo Fisher Scientific). *Ds-NAT1* was excised with *Sal*I from pRK86-*NAT1* and ligated into *Sal*I-linearized pRK401, followed by the exchange of two nucleotides flanking the *Ds-NAT1* 5′-end using primers oKM34 and oKM35, and the Q5 site-directed mutagenesis kit (New England Biolabs, Beverly, MA). The resulting plasmid pRK403a was used for chromosomal integration via homologous recombination of *Ds-NAT1* into the 5′-UTR of the *C. albicans ADE2* gene (Figure 1B, top line).

### Generation of the *MTLa* haploid *Candida albicans* strain GZY896

To isolate a *MTLa* haploid strain from YJB12881 (Hickman *et al.* 2013), single colonies were subjected to repeated rounds of subcultivation in glucose minimal medium supplemented with 40 µg ml^−1^ histidine (GMM + His) medium (Zeng *et al.* 2014). Haploid colonies were identified by flow cytometry analysis and then stored at −80°. Strains were revived on GMM + His plates and several more subcultivation cycles were performed, yielding a haploid strain, GZY892, that appeared stable by flow cytometry. A *ura3*∆ derivative of the strain was constructed by excising the *ura3*Δ::*HIS4* cassette from plasmid pYGS1023 (Hickman *et al.* 2013) and transforming it into GZY892, according to the method of Zeng *et al.* (2014), where it inserted by homologous recombination at the *URA3* locus. The resulting strain was termed GZY896 (*MTLa ura3*∆::*imm434 his4 gal1*∆:: *ura3*∆::*HIS4*).

### Generation of *Ac/Ds* transposable element-carrying *Candida albicans* strains KMY100 and KMY103G1

GZY896 transformation was performed according to the method of Zeng *et al.* (2014). First, linearized pKM300 was used to integrate the *Ac*TPase4xCa expression cassette via homologous recombination into the *NEUT5L* locus (Gerami-Nejad *et al.* 2013) (Figure 1A). Transgenic cells were selected on complete synthetic defined minimal (SDC) plates lacking uridine. In a second step, these transformants and, in parallel, GZY896 cells were transformed with linearized pRK403a to integrate the *Ds-NAT1* into the 5′-UTR of *ADE2* on chromosome 3 (Figure 1B). Transformants were selected by plating the cells on yeast extract peptone (YP) medium + adenine + glucose with 0.4 mg/ml nourseothricin. The resulting strains carrying the *ade2*::*Ds-NAT1* construct alone, or in combination with the *Ac*TPase4xCa expression construct, were termed KMY100 and KMY103G1, respectively.

### Isolation of *Ds* transposants

KMY103G1 cells were grown in SDC + 3% maltose liquid medium at 30° for 24 hr, and then plated on SDC − adenine + glucose plates to select for *ADE2* revertants. To select for reintegration of *Ds-NAT1* elements, *ADE2* revertant colonies were replica-plated on YP + adenine + glucose plates with 0.4 mg/ml nourseothricin and grown for 2 days at 30°.

### Flow cytometry

Flow cytometry was performed as described previously (Hickman *et al.* 2013) using a MACSQuant FLOW cytometer (Miltenyi Biotec GmbH, Bergisch Gladbach, Germany).

### *Ds* excision and reintegration site analysis

*ADE2* revertant colonies were picked, resuspended in 10 µl H_2_O containing 2.5 U Zymolyase (Zymo Research, Irvine, CA) and incubated for 15 min at 30°. Next, 2 µl of a 1:10 dilution of these suspensions was used as template for PCR amplification of *Ds* excision sites with *CaADE2* primers F2 and R2 (Table S1 in File S1) and Q5 DNA Polymerase (New England Biolabs). PCR products were purified on NucleoSpin Gel and PCR Clean-up columns (MACHEREY-NAGEL GmbH, Düren, Germany) and sequenced.

Reintegration sites of *Ds-NAT1* elements were mapped by modified FPNI-PCR (Fusion Primer and Nested Integrated PCR; Wang *et al.* 2011) on *ADE2*^+^-Nat^R^ cells to amplify genomic DNA flanking the *Ds* 3′-end. Target site duplications at three *Ds* reinsertion sites were identified by amplification of novel *Ds-NAT1* insertion sites with pairs of *Ds*-flanking primers (Table S1 in File S1) and sequencing of the PCR products.

### DNA gel blot analysis

Genomic DNAs were isolated from 2 ml cultures of *ADE2* revertants grown in SDC + 2% glucose liquid medium at 30° for 24 hr with the Yeast DNA Preparation Kit (Jena Bioscience GmbH, Jena, Germany). The quality of the isolated DNAs was analyzed on a 1% agarose gel. Next, 200 ng DNA of each sample were digested with 10 U of *Eco*RI (Thermo Fisher Scientific) for 4 hr at 37° and size fractionated on a 0.9% agarose gel overnight at 2 V/cm. The gel was successively soaked for 20 min in 0.2 M HCl, 20 min in 0.5 M NaOH/1.5 M NaCl, and 30 min in 1 M Tris-Cl/1.5 M NaCl pH 7.4. Following overnight capillary transfer of the DNA to a Hybond NX membrane (GE Healthcare, Little Chalfont, UK) the DNA was UV cross-linked (120 J at 254 nm). The membrane was successively prehybridized, hybridized with a Digoxigenin (DIG)-labeled *NAT1* probe, washed, and detected with anti-DIG-AP, Fab fragments, and CDP-*Star* (Roche Applied Science, Penzberg, Germany) following the manufacturer’s protocols. The DIG-labeled *NAT1*-probe was amplified with primers oKM222 and oKM223 using pRK403a as template.

### Data availability

Plasmids and strains are available upon request.

## Results

### An *Ac/Ds* two-component transposon system in *C. albicans*

*C. albicans* belongs to the CUG clade of pathogenic yeasts, in which the trinucleotide CTG specifies serine instead of the commonly encoded leucine (Santos and Tuite 1995). Furthermore, *C. albicans* does not have naturally occurring or engineered plasmids that are maintained autonomously. Therefore, the *Ac*/*Ds* transposon components previously constructed for *S. cerevisiae* (Lazarow *et al.* 2012) were not suitable for application in *C. albicans* and a new *Ac*/*Ds* two-component system for *C. albicans* was required. The system includes a nonautonomous *Ds* element carrying the nourseothricin N-acetyl transferase (*NAT1*) gene as a selectable marker gene, *Ds-NAT1*, and a codon-adapted (“candidized”) hyperactive TPase-coding sequence (*Ac*TPase4xCa) under control of the inducible *CaMAL2* promoter. The original *Ac*TPase4x-coding region with an N-terminally-fused SV40 nuclear localization sequence (NLS) was candidized by replacing two CTG codons with TTG to ensure translation into leucine and by exchanging all codons that are rarely (≤ 11%) used in *C. albicans* with more frequently used codons to ensure efficient translation (Figure S1 in File S1). The *Ac*TPase4xCa expression cassette was inserted into an intergenic region on chromosome 5 (*NEUT5L*; Gerami-Nejad *et al.* 2013) of the haploid *C. albicans* strain GZY896 (Figure 1A). The *Ds-NAT1* element was cloned into the 5′-UTR of *ADE2*, 26-bp upstream of the *ADE2* start codon on chromosome 3 (Figure 1B), and recombined into GZY896 with or without the *Ac*TPase4xCa gene, respectively. The resulting strains KMY100 (*ade2*::*Ds-NAT1*) and KMY103G1 (*Ac*TPase4xCa::*NEUT5L ade2*::*Ds-NAT1*) grow a little slower than *ADE2* cells and form red colonies on SDC medium.

### *Ac*TPase4xCa expression induces *Ds-NAT1* excision

The stability of the *Ds-NAT1* insertion in the *ADE2* 5′-UTR was examined by propagating KMY100 cells for four passages on YP + adenine + glucose medium. After each passage, we plated three colonies and observed only red colonies, suggesting that the *ade2*::*Ds-NAT1* locus is stable. Accordingly, amplification of the *Ds-NAT1* from cells of each passage with flanking *ADE2*-primers yielded a single 2.6-kb band that was indistinguishable from that obtained from cells after the first passage (Figure 1C), indicating that the *Ds-NAT1* insertion remained stably inserted in the *ADE2* promoter.

Similarly, KMY103G1 cells grew as red colonies on SDC plates after propagation for 24 hr in noninducing SDC + adenine + glucose liquid medium. In contrast, when cells were precultured in glucose-free SDC + adenine + maltose medium to induce *Ac*TPase4xCa expression, white *ADE2* revertant colonies appeared with a frequency ranging from 0.0001 to 0.001%.

**Figure 2:**
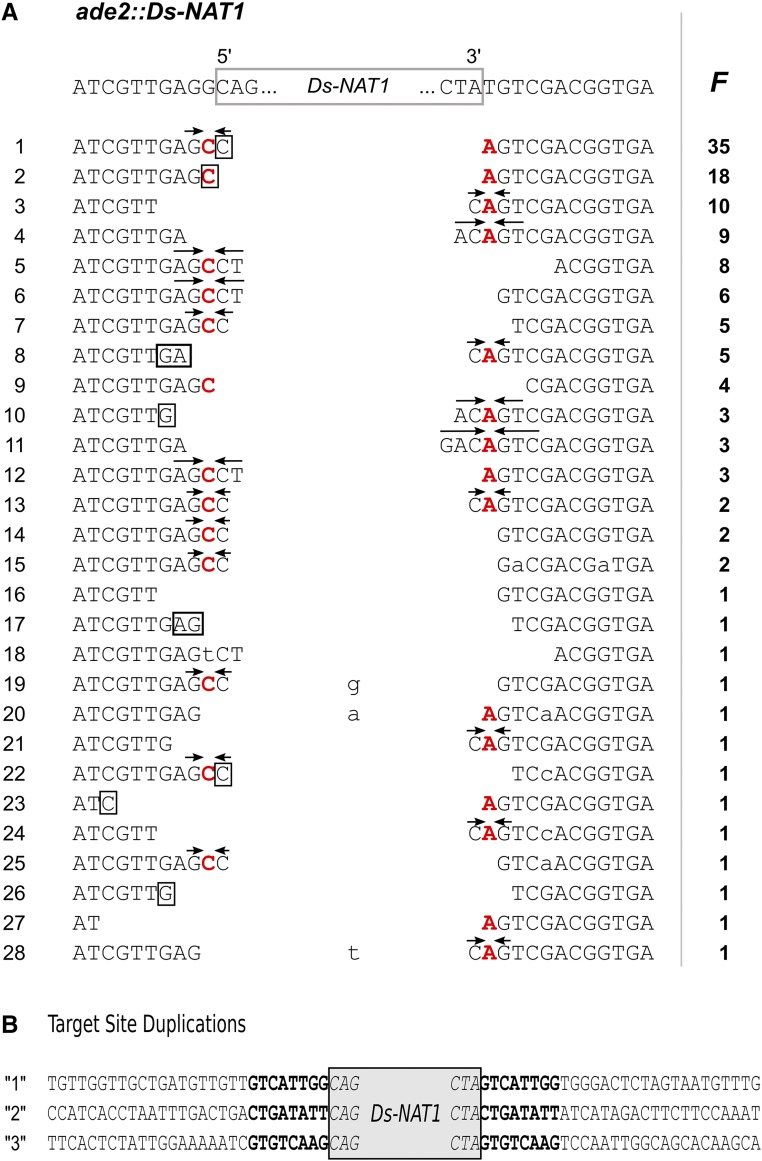
DNA sequences at the *Dissociation* (*Ds*)*-NAT1* excision and reinsertion sites. (A) *Ds* excision footprints of 128 independent KMY103G1 *ADE2* revertant colonies. The top row shows the *ade2*::*Ds-NAT1* sequence on chromosome 3. Rows 1–28 show the recovered *Ds* excision footprints and their incidence (*F*) in independent *ADE2* revertants from *Ac*TPase4xCa-expressing KMY103G1 cells grown in maltose-containing medium. Arrows above the sequences indicate inverted repeats centered around the complementary bases C and A (boldface red letters) of the nucleotides bordering the *Ds-NAT1* transposon that result from the resolution of intermediate hairpin structures. Lower case letters indicate nucleotides that are not explained with the hairpin model. (B) Target site duplications at *Ds-NAT1* reinsertion sites in transposants “1,” “2,” and “3”; see Table S2 in File S1.

From 128 independent *ADE2* revertant KMY103G1 colonies, the *Ds-NAT1* excision sites were amplified. The PCR bands from all revertants were indistinguishable in size from the 510-bp band in the progenitor strain GZY896, suggesting that the *Ds-NAT1* had been excised from the *ADE2* promoter locus (Figure 1, D and E). Sequencing of the PCR bands revealed 28 different *Ds* excision footprints (Figure 2A). Two predominant footprints, which differed in a single nucleotide at the fusion site, were recovered in 35 and 18 clones, respectively. In addition to these two major footprints, 26 other, different footprints were observed less frequently, with half of them appearing only once. Despite the limited number of footprints analyzed, the bias in excision site repair products was obvious. All footprints exhibited short deletions or palindromic insertions (also termed P-nucleotide insertions; Huefner *et al.* 2011) at either side of the breakpoint, which closely resembled the *Ac*/*Ds* excision footprints in plants and in budding yeast.

### *Ds-NAT1* efficiently reinserts into the genome

Reintegration of the *Ds-NAT1* transposon was investigated by cultivating 10 individual red KMY103G1 colonies in SDC + adenine + maltose liquid medium and spreading aliquots on SDC − adenine + glucose plates, yielding 1722 *ADE2* revertant colonies. The white *ADE2* colonies were replica-plated onto nourseothricin-containing SDC − adenine + glucose plates and grown for 2 days at 30°. In total, 1528 colonies (∼90%) grew on nourseothricin. This unexpectedly high frequency (in plants and *S. cerevisiae* reinsertion frequencies of ∼60% were observed) prompted us to scrutinize the ploidy of the cells. All five KM103G1 colonies that we tested by flow cytometry were diploid. A DNA gel blot analysis of genomic DNA from white *ADE2* revertant colonies revealed that, with only one exception, all clones still contained the *ade2*::*Ds-NAT1* allele (Figure 1F). Fourteen clones exhibit an additional, differently sized band, indicating reinsertion of the *Ds-NAT1* transposon elsewhere in the genome. One exceptional clone showed no *ade2*::*Ds-NAT1* band, with one smaller and one larger band. These data demonstrate that: (i) the KMY103G1 progenitor cells had undergone autodiploidization prior to transposon mobilization, (ii) the *Ds-NAT1* transposon had excised from only one of the two alleles with the exception of one clone, and (iii) in 15 of the 28 *ADE2* revertant clones, the transposon reintegrated in different positions in the *Candida* genome. In the one exceptional clone (marked with a red asterisk), the *Ds-NAT1* transposons apparently transposed from both alleles and reintegrated in two novel chromosomal sites.

For three *ADE2* revertant clones, the genomic reinsertion sites were identified by FPNI-PCR of the 3′-end of *Ds-NAT1* (Figure 2B and Table S2 in File S1). Two of the *Ds-NAT1* elements reintegrated in chromosome 3, 174- and 266-kb distal and proximal to the *ADE2* locus, respectively. The third element transposed to chromosome 5. Amplification and sequencing of both *Ds-NAT1* flanking sequences revealed the canonical 8-bp target site duplications generated upon integration of the *Ds-NAT1* transposable elements (Figure 2B). Thus, transposition occurred, both close to the original insertion site as well as on a different chromosome.

## Discussion

Approaches for genome-wide, untargeted insertion mutagenesis of eukaryotic genomes are frequently based on chemical or ballistic transformation with selectable DNA marker molecules [in fungi, sometimes boosted by restriction enzyme-mediated integration (REMI) reviewed in Jiang *et al.* (2013)], on *A. tumefaciens* infection and T-DNA insertion in plants, or by transposon tagging. In micro-organisms that can be efficiently transformed, large mutant collections can be generated by transformation with plasmid-based mutant libraries produced by *in vitro* transposition of bacterial transposons (see for example Kumar *et al.* 2004). However, transformation is inefficient in the majority of eukaryotes. This limitation can be avoided by using *in vivo* transposon tagging that exploits endogenous or heterologous transposons. The advantage of using a heterologous transposon is that it will be unique in the genome and is easily detected. The diploid *C. albicans* WO-1 genome is composed of 2.1% retroelements and contains only two copies each of a Tc1/mariner-like DNA transposon and a (defective) Mutator-like element (Butler *et al.* 2009). However, endogenous transposition activity has not been reported for any of these elements, despite a report that *C. albicans* transposons are not modified by DNA methylation (Mishra *et al.* 2011). In this study, we explored the suitability of the maize *Ac*/*Ds* transposons for *in vivo* transposon insertion mutagenesis in *C. albicans*.

Like in all heterologous host organisms tested, the nonautonomous *Ds* element in *C. albicans* is genetically stable and its mobilization strictly depends on expression of the *Ac* TPase. The *Ds* excision footprints display sequence features similar to those that have been reported for *Ac*/*Ds* in other plants and in *S. cerevisiae* (Lazarow *et al.* 2012): deletions and palindromic P-nucleotide insertions at the repaired DNA joint are centered around the complement of the base adjacent to the transposon ends. This indicates that in haploid *C. albicans*, the DNA double-strand break (DSB) repair pathway forms a DNA hairpin followed by hairpin resolution and nonhomologous end joining repair of the empty donor sites, essentially as has been suggested for plants and *S. cerevisiae* (Coen *et al.* 1986; Lazarow *et al.* 2012). However, an interesting distinction is that the structural heterogeneity of the footprints in *C. albicans* is lower than in *Arabidopsis* and budding yeast. For example, the longest P-nucleotide insertions were three nucleotides and occurred only in a single footprint type three times among the 128 footprints (2.3%) (Figure 2A, line 11), whereas in *S. cerevisiae*, the maximal palindrome arm length was 20 nucleotides and 66 of 140 footprints (47%) contained palindromes with ≥3 nucleotide-long arms (Lazarow *et al.* 2012). Also in *Arabidopsis*, the frequency of palindromes at least three nucleotides in length was much higher than in *C. albicans* (20–30%) (Huefner *et al.* 2011; Lazarow *et al.* 2012). The shorter palindrome lengths in *C. albicans* indicate that the (unknown) hairpin-opening enzymes introduce nicks closer to the apex of the hairpin than in *S. cerevisiae* and plants. In *S. cerevisiae*, mutations in the Artemis-related Pso2 protein, which cleaves DNA hairpins (Tiefenbach and Junop 2012), reduced *Ds* excisions 10-fold and increased atypical *Ds* excision site repair products (Yu *et al.* 2004). It will be interesting to determine if the *C. albicans* and *S. cerevisiae PSO2* orthologs have different activities, or if other enzymes are responsible for the distinct footprints and excision frequencies.

*Ac/Ds* excision footprints in both *S. cerevisiae* and *A. thaliana* are accompanied by putative microhomologies (MHs) of the repair intermediates after DNA hairpin opening (Yu *et al.* 2004; Huefner *et al.* 2011; Lazarow *et al.* 2012). The frequency of footprints with MHs ranged from 85 to almost 100% in wild-type *A. thaliana* (Huefner *et al.* 2011; Lazarow *et al.* 2012) and from 88 to 89% in *S. cerevisiae* (Yu *et al.* 2004; Lazarow *et al.* 2012). In contrast, only 51% of the footprints in *C. albicans* had putative MHs. This suggests that DNA hairpin opening and DSB repair proceeds somewhat differently in *C. albicans* than in the model yeast *S. cerevisiae* and plants. Shorter P-nucleotide insertions in *C. albicans* may indicate that the nuclease that binds to and opens DNA hairpins is more stringently locked to the hairpin apex in *C. albicans* than the functional homologs in plants and budding yeast. While MHs are not essential for the fusion of open ends, terminal MH in double-stranded DNA with short 3′ overhanging single strands stabilizes DSB repair intermediates and enhances the efficiency of repair (Gu *et al.* 2007). Since DNA ligase IV (encoded by *LIG4*) is critically involved in the repair of DSBs in all eukaryotes and also can ligate DNA ends lacking MHs (Gu *et al.* 2007), the lower frequency of MHs at *Ds* excision footprints suggests that *LIG4*-dependent DSB repair is likely to be more active in *C. albicans* than in the other organisms tested.

The high efficiency of the *Ac*/*Ds* transposon system demonstrated in this study makes it a potentially superior molecular tool for genome-wide *in vivo* insertion mutagenesis in *C. albicans* compared to alternative approaches. Several *in vitro* insertion mutant libraries were constructed in diploid *C. albicans* using bacterial *Tn*7 or *Tn*5 derivatives that were transposed into isolated genomic DNA fragments, cloned, amplified in *Escherichia coli*, and then transformed into *C. albicans*, where the transposon together with flanking genomic DNA integrated by homologous recombination into the chromosomes (Uhl *et al.* 2003; Oh *et al.* 2010; Bharucha *et al.* 2011). However, this approach was labor-intensive and yielded 18,000, 3633, and 6528 heterozygous transformants, respectively, in the three studies. Thus, the *in vitro* approach is much less practical for repeated application in multiple different strains, for example clinical isolates. Another advantage of the transposon system described here is that, in addition to generating total loss-of-function alleles, it should be useful to identify haploinsufficient genes in diploid cells and other conditionally essential genes, as was recently demonstrated for *Ac/Ds* insertions in *S. cerevisiae* (Michel *et al.* 2017). Furthermore, it can easily be modified for activation-tagging applications, as has been demonstrated in plants [reviewed in Lazarow *et al.* (2013)], as well as for the generation of overexpression and epitope tagging libraries. The beauty of the system is the ease in generating new libraries with thousands, to hundreds of thousands, of new mutants in a single strain background. When coupled with deep sequencing technologies, this provides the potential for facile, rapid generation of large libraries of random insertion mutants without the need for efficient transformation frequencies.

## Supplementary Material

Supplemental material is available online at www.g3journal.org/lookup/suppl/doi:10.1534/g3.117.300388/-/DC1.
