# Supplementary material for: Maize Transposable Elements Ac/Ds as Insertion Mutagenesis Tools in Candida albicans
Source: G3 (Bethesda). 2018 Jan 29;8(4):1139–45. doi: 10.1534/g3.117.300388 (PMC5873905; doi:10.1534/g3.117.300388)
Supplement: Supplementary file 1 [file 1139FileS1.pdf]

Maize Transposable Elements *Ac*/*Ds* as Insertion Mutagenesis Tools in *Candida albicans*

Kevin Mielich, Ella Shtifman-Segal, Julia C. Golz, Guisheng Zeng, Yue Wang, Judith Berman, Reinhard Kunze

Supplemental Data

```
ATG GGG CCA AAA AAG AAG AGA AAG GTG GCC ATG GCT ATT GTT CAT GAA CCA CAA CCA CAA CCA CAA CCA CAA CCA GAA CCA CAA CCA CAG  < 90
M   G   P   K   K   K   R   K   V   A   M   A   I   V   H   E   P   Q   P   Q   P   Q   P   Q   P   E   P   Q   P   Q

CCA CAA CCT GAA CCA GAA GAA GAA GCA CCA CAG AAG AGA GCA AAG AAG TGC ACA TCA GAT GTA TGG CAG CAT TTC ACC AAG AAG GAA ATT  < 180
P   Q   P   E   P   E   E   E   A   P   Q   K   R   A   K   K   C   T   S   D   V   W   Q   H   F   T   K   K   E   I

GAA GTG GAG GTC GAT GGA AAG AAA TAC GTT CAG GTA TGG GGA CAT TGC AAC TTT CCT AAT TGC AAG GCT AAG TAT AGA GCT GAG GGT CAT  < 270
E   V   E   V   D   G   K   K   Y   V   Q   V   W   G   H   C   N   F   P   N   C   K   A   K   Y   R   A   E   G   H

CAT GGA ACA TCT GGA TTT CGA AAT CAC TTG AGA ACA TCA CAT AGT TTA GTT AAA GGT CAG TTG TGT TTA AAA AGT GAA AAG GAT CAT GGA  < 360
H   G   T   S   G   F   R   N   H   L   R   T   S   H   S   L   V   K   G   Q   L   C   L   K   S   E   K   D   H   G

AAA GAC ATA AAT TTG ATT GAG CCT TAT AAG TAC GAT GAA GTG GTT TCT TTA AAG AAG TTA CAT TTG GCA ATA ATC ATG CAT GAA TAT CCT  < 450
K   D   I   N   L   I   E   P   Y   K   Y   D   E   V   V   S   L   K   K   L   H   L   A   I   I   M   H   E   Y   P

TTC AAT ATT GTA GAA CAT GCT TAC TTT GTT GAG TTT GTT AAG TCT TTG AGA CCT CAC TTT CCA ATA AAG TCA CGT GTC ACT GCT AGA AAA  < 540
F   N   I   V   E   H   A   Y   F   V   E   F   V   K   S   L   R   P   H   F   P   I   K   S   R   V   T   A   R   K

TAT ATC ATG GAT TTG TAT TTG GAA GAA AAA GAA AAG TTG TAT GGA AAA TTA AAA GAT GTT CAG TCT AGA TTC AGT ACA ACT ATG GAT ATG  < 630
Y   I   M   D   L   Y   L   E   E   K   E   K   L   Y   G   K   L   K   D   V   Q   S   R   F   S   T   T   M   D   M

TGG ACA TCT TGT CAA AAT AAG TCA TAC ATG TGT GTC ACC ATC CAT TGG ATT GAT GAT GAT TGG TGT TTG CAA AAA AGA ATT GTT GGA TTT  < 720
W   T   S   C   Q   N   K   S   Y   M   C   V   T   I   H   W   I   D   D   D   W   C   L   Q   K   R   I   V   G   F

TTT CAT GTT GCA GGG AGA CAC ACT GGA CAA AGA TTA TCA CAA ACC TTC ACT GCA ATC ATG GTT AAG TGG AAC ATT GAG AAA AAA TTG TTT  < 810
F   H   V   A   G   R   H   T   G   Q   R   L   S   Q   T   F   T   A   I   M   V   K   W   N   I   E   K   K   L   F
                                                 BsrGI
GCC TTG TCT TTG GAT AAT GCT AGT GCA AAT GAA GTA GCT GTA CAC GAT ATA ATT GAG GAT TTG CAG GAC ACT GAT TCA AAT TTA GTT TGT  < 900
A   L   S   L   D   N   A   S   A   N   E   V   A   V   H   D   I   I   E   D   L   Q   D   T   D   S   N   L   V   C

GAT GGT GCT TTC TTT CAT GTG AGA TGT GCT TGT CAC ATA TTG AAC TTG GTT GCA AAG GAT GGA TTG GCT GTA ATT GCA GGA ACA ATT GAG  < 990
D   G   A   F   F   H   V   R   C   A   C   H   I   L   N   L   V   A   K   D   G   L   A   V   I   A   G   T   I   E

AAA ATC AAA GCT ATT GTT TTA GCT GTA AAA TCT TCT CCT TTG CAG TGG GAA GAA TTA ATG AAG TGT GCT AGT GAA TGT GAC TTG GAT AAA  < 1080
K   I   K   A   I   V   L   A   V   K   S   S   P   L   Q   W   E   E   L   M   K   C   A   S   E   C   D   L   D   K

TCT AAA GGG ATC TCA TAT GCT GTC TCA ACT AGA TGG AAT TCA ACC TAT TTG ATG TTG AGA GAT GCC TTA TAT TAT AAG CCT GCA TTA ATA  < 1170
S   K   G   I   S   Y   A   V   S   T   R   W   N   S   T   Y   L   M   L   R   D   A   L   Y   Y   K   P   A   L   I

AGA TTA AAA ACA AGT GAT CCT AGA AGA TAC GAT GCA ATT TGT CCT AAA GCC GAG GAG TGG AAG ATG GCA TTA ACT TTA TTT AAG TGT TTG  < 1260
R   L   K   T   S   D   P   R   R   Y   D   A   I   C   P   K   A   E   E   W   K   M   A   L   T   L   F   K   C   L

AAG AAG TTT TTT GAT TTG ACT GAA TTG TTA TCT GGT ACT CAA TAT TCA ACT GCA AAT TTA TTT TAC AAA GGT TTC TGT GAG ATA AAG GAT  < 1350
K   K   F   F   D   L   T   E   L   L   S   G   T   Q   Y   S   T   A   N   L   F   Y   K   G   F   C   E   I   K   D

TTG ATT GCC CAA TGG TGT GTT CAT GAA AAA TTT GTC ATT AGA AGA ATG GCC GTT GCA ATG AGT GAA AAG TTT GAG AAA TAT TGG AAA GTG  < 1440
L   I   A   Q   W   C   V   H   E   K   F   V   I   R   R   M   A   V   A   M   S   E   K   F   E   K   Y   W   K   V
                              SphI
TCT AAT ATT GCA TTA GCT GTA GCA TGC TTC TTA GAC CCT AGA TAC AAG AAA ATA TTG ATT GAG TTC TAT ATG AAA AAA TTT CAT GGT GAT  < 1530
S   N   I   A   L   A   V   A   C   F   L   D   P   R   Y   K   K   I   L   I   E   F   Y   M   K   K   F   H   G   D

TCA TAC AAA GTT CAT GTA GAT GAC TTT GTT AGA GTC ATT AGA AAA TTG TAT CAA TTC TAT TCT AGT TGT AGT CCT TCA GCT CCA AAG ACA  < 1620
S   Y   K   V   H   V   D   D   F   V   R   V   I   R   K   L   Y   Q   F   Y   S   S   C   S   P   S   A   P   K   T

AAG ACA ACT ACT AAT GAT AGT ATG GAT GAT ACC TTG ATG GAA AAT GAA GAT GAT GAA TTT CAA AAC TAT TTG CAT GAG TTG AAG GAT TAT  < 1710
K   T   T   T   N   D   S   M   D   D   T   L   M   E   N   E   D   D   E   F   Q   N   Y   L   H   E   L   K   D   Y

GAT CAA GTA GAG TCA AAT GAA TTG GAT AAA TAT ATG TCT GAA CCA TTA TTG AAG CAT AGT GGT CAG TTT GAT ATT TTA TCA TGG TGG AGA  < 1800
D   Q   V   E   S   N   E   L   D   K   Y   M   S   E   P   L   L   K   H   S   G   Q   F   D   I   L   S   W   W   R

GGA AGA GTT GCA GAA TAT CCT ATT TTG ACC CAA ATT GCA AGA GAT GTG TTA GCA ATA CAA GTG TCA ACT GTT GCT TCT GAG TCT GCT TTC  < 1890
G   R   V   A   E   Y   P   I   L   T   Q   I   A   R   D   V   L   A   I   Q   V   S   T   V   A   S   E   S   A   F

AGT GCT GGT GGT CGT GTT GTT GAT CCT TAC AGA AAT CGT TTA GGT TCA GAG ATT GTT GAA GCT TTG ATA TGC ACA AAA GAT TGG GTA GCA  < 1980
S   A   G   G   R   V   V   D   P   Y   R   N   R   L   G   S   E   I   V   E   A   L   I   C   T   K   D   W   V   A

GCA TCT AGA AAA GGT GCT ACA TAT TTT CCA ACA ATG ATT GGT GAT TTG GAG GTG TTA GAC TCT GTT ATT GCT GCT GCA ACA AAT CAT GAG  < 2070
A   S   R   K   G   A   T   Y   F   P   T   M   I   G   D   L   E   V   L   D   S   V   I   A   A   A   T   N   H   E

AAT CAT ATG GAT GAG GAT GAA GAC GCA ATA GAA TTT TCT AAG AAT AAT GAA GAT GTA GCA AGT GGA TCA TCT CCA TGA  < 2148
N   H   M   D   E   D   E   D   A   I   E   F   S   K   N   N   E   D   V   A   S   G   S   S   P   *
```

Supplemental Figure S1: Sequence of the *Ac*TPase4xCa coding region. The *Ac*TPase4xCa open reading frame is based on the *Ac*TPase4x sequence (Lazarow et al., 2012). It lacks the N-terminal 102 amino acids of the original *Ac*TPase, carries four amino acid substitutions (brown letters) and is N-terminally extended by the SV40 nuclear localization sequence (green box). Codons highlighted in blue were modified in the original SV40 NLS and *Ac*TPase4x sequence to optimize codon usage for protein expression in *C. albicans*. Two CTG leucine codons in the original sequence were exchanged by TTG (yellow boxes). The highlighted *Bsr*GI and *Sph*I restriction sites are lacking in the original sequence.

Supplemental Table S1. Primer sequences

Primers for cloning of *Ds-NAT1* and *Ac*TPase$_{4x}$Ca

Primer	Sequence
oTEFmut-F	GCGGGTCGACACTGGATGGCGG
oTEFmut-R	CCAAGTCGACGACATGGAGGCCCA
oKM34	CTTGTATCGTTGAGGCAGGGATGAAAG
oKM35	CACTACACATAATGGATAGCAAAAC
oMAL2-F	CCGCGGTGACCTTTTTGTCTAGTACCATC
oMAL2-R	GCGGTTCGAATGTAGTTGATTATTAGTT
oADHT-F	GCGGGCTAGCTCTTGGACACCAGTAAAT
oADHT-R	CCGCGGTCACCCAACTGTATAAGATAGTA
oKM222	GTAGCCAAACCCATCAAAGCTCTACC
oKM223	GACATGGAGGCCCAGAATACCC

Primers for identification of footprints

Primer	Sequence
R2 (oCaADE2-up)	CCGCATCAACATCAACATG
F2 (oCaADE2-low)	GCTGAATCCACGACAAATAACG
F1 (oKM214)	GTTTTTGGAGTATTGTGATGGTAT
R1 (oKM215)	CCAAACTTTTCCCCAATGTGTAAC

Primers for FPNI-PCR

round	Forward primer	Reverse primer
1st	CCGTTCGTTTTCGTTACCGGTATATC	CGCAGGTGACATAGATGC
2nd	CCGTCCCGCAAGTTAAATATG	GACATAGATGCTTAGCGCTGAGG
3rd	GTATTTTACCGACCGTTACCGACC	CGCAGGTGACATAGATGCTTAGCGCTGAGGNNNNNNNNN
TSD1	TCCCTAAATTCTCTGTGCCCC	TGGTGGTTGCTGGAGTTGTT
TSD2	TTCCAGTGGTGCGAGGTAAA	TGACGGATAGCGGAACCAAA
TSD3	TGGATCCCCGTCATTCCTCT	GGTCAGCTATGGGCAAACCT

Supplemental Table S2: Reinsertion sites of three *Ds-NAT1* transposition events

Transposant no.	Chromosome	Reinsertion site	ORF annotation
**1**	3	orf19.5992 (position 1118783)	(orf19.5992) Zn(II)2Cys6 transcription factor; regulator of white-opaque switching; required for maintenance of opaque state; Hap43-induced
**2**	3	between orf19.319 and orf19.320 (position 679408)	
**3**	5	orf19.6679 (position 813686	uncharacterized; (orf19.6679) Ortholog(s) have cytosol, nucleus localization
